# Indication of nerve growth factor binding components from herbal extracts by HerboChip: a platform for drug screening on a chip

**DOI:** 10.1186/s13020-016-0107-8

**Published:** 2016-07-23

**Authors:** Pinky Sum Chi Lee, Laura Minglu Zhang, Artemis Lu Yan, Kelly Yin Ching Lam, Tina Tingxia Dong, Huangquan Lin, Gallant Kar Lun Chan, Karl Wah Keung Tsim

**Affiliations:** Division of Life Science and Center for Chinese Medicine R&D, The Hong Kong University of Science and Technology, Hong Kong SAR, China

## Abstract

**Background:**

HerboChip is an array of different fractions deriving from herbal extracts. This study aimed to identify effective components from Chinese medicine (CM) that interact with nerve growth factor (NGF) as a target using HerboChip.

**Methods:**

Fifty types of CM that are traditionally used as remedies for emotion imbalance were selected and extracted with 50 % ethanol. Biotinylated-NGF was hybridized with over 300 chips coated with different HPLC-separated fractions from CM extracts and straptavidin-Cy5 was used to identify the NGF-bound fractions.

**Results:**

Over 300 chips were screened within a week, and 17 positive hits were identified. The interaction of the identified herbal extracts with NGF was confirmed in cultured PC12 cells. Co-application of NGF and herbal extract interfered with NGF-induced expression of neurofilaments, including NF68 and NF200 in cell cultures. Western blot analysis comparing the intensity of phosphorylated cAMP response element-binding protein (CREB) over total CREB showed NGF-induced CREB phosphorylation was modulated by the identified herbal extracts. Five CM herbs showed activating activities on the NGF response and nine CM herbs showed inhibiting activities.

**Conclusion:**

The current result supported the applicability of HerboChip for screening NGF binding components from herbal extracts.

**Electronic supplementary material:**

The online version of this article (doi:10.1186/s13020-016-0107-8) contains supplementary material, which is available to authorized users.

## Background

Chinese medicine (CM) is considered effective for treating diseases and improving health in China and Asia [1–3]. However, many of the active ingredients of CMs are still not fully identified. Traditional methods to isolate active ingredients are time-consuming and have a limited success rate [4]. Herein, we aimed to identify active compounds from CMs for drug development against diseases by using a high-throughput analytic platform, HerboChip (Kunming, Yunnan, China) [5].

The initial amount of CM required for traditional screening methods is large. The scale of preparation to isolate compounds using column chromatography must be large enough to obtain a sufficient amount of the extract for cell-based studies. The HerboChip platform requires small amounts of CMs, saving resources. HerboChip requires only small amounts of CM extracts for HPLC fractionation and chip dotting [6]. The HerboChip screening method employs small volumes of reagents, and takes several hours for hybridization and signal detection [6].

Nerve growth factor (NGF) is a neurotrophic factor relating to the causes of neurodegenerative diseases such as depression and Alzheimer’s disease [7]. Treatment with NGF can restore certain aspects of memory functions in animals [8]. Anti-NGF drugs are being developed as pain killers for chronic diseases [9]. In view of NGF being the known disease target, binding between herbal fractions on the chip and NGF can identify potential targets for drug development of treatments for neurodegenerative diseases.

To achieve this, the known disease target NGF should be immobilized on HerboChip and used to screen herbal compounds. Three major steps are involved to identify potential drug leads: (1) HPLC fractionation of herbal extract; (2) dotting fractionated extract on a chip; and (3) the screening process. The first step is time-consuming but can be used to prepare chips that can be stored for over a year at low temperature. The first target using this system was cytochrome P450 3A4 (CYP3A4). CYP3A4 acts as a protein substrate in enzymatic reactions. Inhibition of CYP3A4 enhanced the effectiveness of saquinaviras, an anti-retroviral drug used in HIV therapy [10]. HerboChip screening reviled *Sophora flavescens* exhibited the maximal inhibition towards CYP3A4 [5]. Another successful application of the HerboChip screening platform was published by Huang et al. [6] in 2015. *Geranium wilfordii*, a renowned CM for treating rheumatoid arthritis by an anti-inflammatory mechanism, was demonstrated to target TNF-alpha. The HerboChip platform served as a tool to elucidate the mechanism of the CM in this study rather than identify potential leads in a horizontal screening exercise.

This study aims to identify active components from CMs using HerboChip with NGF as the target.

## Methods

### Preparation of HerboChip and herbal extracts

HerboChip arrays were purchased from Yannanbaiyao Group Tianzihong Pharmaceutical Co. Ltd (Kunming, Yunnan, China). Herbal extracts were prepared by extracting 50 g of herb powder with 1 L of ethanol for 3 days, and then concentrating the ethanoic extract to 30 mL. The herbs were collected in the field or purchased from commercial sources and morphologically authenticated by Xiaoyuan Yang at the Yunnan Institute of Materia Medica according to the Chinese Pharmacopoeia 2010 [11]. Voucher specimens of the herbs were sent to Yannanbaiyao Group Tianzihong Pharmaceutical Co. Ltd. to be cataloged. The herbs were tested for qualification according to the guidelines of the Chinese Pharmacopeia [11]. Herbal extracts were lyophilized and stored in vacuum. Extracts were re-suspended in 50/50 water/ethanol and fractionated by a standardized HPLC method in gradient mode with a duration of 96 min (0 min; 0 % acetonitrile → 96 min; 100 % acetonitrile). Fractions were collected according to retention time with each fraction corresponding to 1 min. The chip surfaces were activated and had the epoxy groups exposed before dotting. The herbal fractions were dotted and fixed on the surface of activated chips by an automatic arrayer (Biodot A101, Shuai Ran Precision, Taoyuan, Taiwan). Standardized herbal extracts were provided by Herbocopoeia Pharmaceutical Inc. The lyophilized standards were re-dissolved with DMSO at a concentration of 100 mg/mL as a stock for cell culture studies.

### Screening of HerboChip

Biotinylated-NGF was prepared using an EZ-Link™ NHS-PEG4-Biotinylation kit (Thermo Fisher scientific, Rockford, IL, USA). About 0.5 mg of NGF (Alomone Labs, Israel) was dissolved in 0.5 mL of phosphate-buffered saline (PBS), mixed with 39 μL of 20 mM biotin solution and incubated at room temperature for 1 h. Excess biotin reagent was removed by a desalting column. The labeled NGF was stored at −80 °C. According to traditional Chinese medicinal theory, around 300 types of CM are related to emotional calmness. These CMs were selected and screened with the biotinylated-NGF probe. The biotinylated-NGF was hybridized with HerboChip dotted with different herbal fractions. Streptavidin-Cy5™ (Invitrogen Life Technologies) was used to detect biotinylated-NGF on the HerboChip arrays. The fluorescence signal of Streptavidin-Cy5 was measured at 535 nm by a fluorophore microarray scanner (GenePix 4100A, Molecular Devices Corp., CA, USA). The fluorescence results were analyzed with GenePix Pro 7 (ver. 7.1.16) software provided by the manufacturer of the microarray scanner (Silicon Valley, CA, USA). Fluorescence intensity higher than 600 was counted as positive.

### Cell culture

Pheochromocytoma PC12 cells, a cell line derived from rat adrenal medulla, were obtained from the American Type Culture Collection (ATCC^®^ CRL-1721™). PC 12 cells were maintained in Dulbecco’s modified Eagle’s medium supplemented with 6 % fetal calf serum, 6 % horse serum, 100 units/mL of penicillin, and 100 μg/mL of streptomycin (Invitrogen) in a humidified CO_2_ (7.5 %) incubator at 37 °C. Culturing media were renewed every 2–3 days. Cell viability was assessed by 3-(4,5-dimethyl-2-thiazolyl)-2,5-diphenyl-2H-tetrazolium bromide (MTT; Sigma) assay to decide the working concentration of each CM extract [9]. Cell cultures were challenged with herbal extracts (10, 30 and 100 μg/mL) and MTT solution was added and incubated for 1 h at 37 °C. The absorbance of the cell cultures were measured at 570 nm in a microplate reader (Thermo Scientific). The protocols to measure neurite out growth and neurofilament expression have been described previously [12].

### cAMP response element binding protein phosphorylation

PC 12 cells were starved for 5 h and then challenged with NGF and CM extract for 0, 2 or 5 min. Kinase Inhibitor K252a (Sigma, purity ≥98 %) served as a positive control that would block the TrkA receptor at one of the NGF binding sites. Cells were solubilized in lysis buffer containing 125 mM Tris-hydrochloride (pH 6.8), 4 % sodium dodecyl sulfate (SDS), 20 % glycerol and 2 % 2-mercaptoethanol and stored frozen at −20 °C. Lysates were separated on 8 % SDS–polyacrylamide gels and transferred to a nitrocellulose membrane. The membrane was blocked with 5 % non-fat milk and then incubated with anti-phospho-cAMP response element binding protein (CREB) primary antibodies (1:5000; Cellular Signaling Technology) overnight, followed by anti-rabbit secondary antibodies (1:5000; Invitrogen) for an hour. The immune complexes were visualized by the enhanced chemiluminescence (ECL) method (GE Healthcare). The control and samples were run on the same gel under strict standardized ECL conditions and the intensities of the bands were compared using an image analyzer and ImageJ 1.48v (NIH, USA) software [13].

### DNA transfection and luciferase assay

The DNA constructs of pNF-68-Luc and pNF-200-Luc reporter genes in a pLightSwitch_Prom vector were purchased from Active Motif. Cultured PC 12 cells were transiently transfected with pNF-68-Luc and pNF-200-Luc by lipofectamine 3000 reagent (Invitrogen) [13]. The transfected cells were exposed to the NGF and CM treatment and a luciferase assay was performed using a Dual-light^®^ System (Applied Biosystems, CA, USA) with K252a as a positive control. The transfected cells were lysed with phosphate lysis solution at pH 7.8 with 0.2 % triton and centrifuged at 16,000×*g* at 4 °C for 10 min (Eppendorf, Hamburg, Germany). The cell lysates were transferred to 96-well assay plate and set on the GloMax™ 96 Microplate Luminometer (Promega, UK). The intensities of each sample were normalized using protein concentration.

### Statistical analysis

The protein concentration was measured using the Bradford’s method (Bio-Rad Laboratories, Singapore). All data were analyzed using one-way analysis of variance followed by Student’s *t* test (GraphPad Prism 5 (ver 5.01), GraphPad Software, CA, USA). Results were classed into three levels of statistical significance: * where *P* < 0.05; ** where *P* < 0.01 and *** where *P* < 0.001.

## Results

**Fig. 1:**
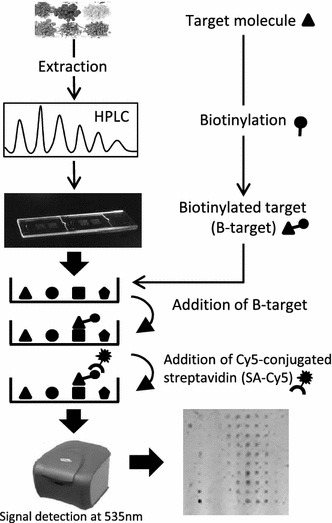
HerboChip screening protocol. Herbal extracts were fractionated by HPLC and then dotted and fixed on a chip. The chip was hybridized with biotinylated protein target (NGF), and then with straptavidin-Cy5 before fluorescence detection

**Fig. 2:**
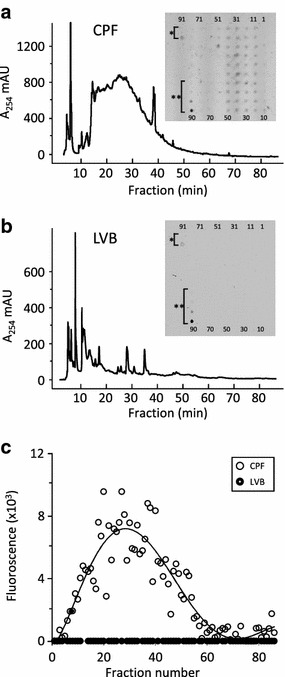
The read out of HerboChip screening. HPLC chromatograms and HerboChip screening images of **a** Crataegi Pinnatifidae Fructus (CPF), **b** Lili Virduli Bulbus (LVB) and **c** the fluorescence signal converted profile from HerboChip of CPF and LVB. The number corresponds to the HPLC fraction number and the dotted chip number. *Asterisk* represents the positive control (straptavidin-Cy5) and the negative control (fixing solution) of the chip. *Double asterisk* represents the array control dotted with an increasing concentration of biotin. A representative profile is shown, *n* = 5

Biotinylated-NGF was used to screen HerboChip arrays coated with HPLC-separated fractions from CM extracts using the procedure shown in Fig. 1. About 300 CMs reported to have effects that treat emotion imbalance were chosen for screening. Each CM was tested in five replicates to ensure consistency. Seventeen CM extracts gave positive hits in our first screening. The sources of those extracts, as well as their abbreviations, and their signal intensity are given in Table 1. The position of each signal was correlated to the fractionation time of the HPLC separation. Chip images, fluorescence signals, converted profiles and the corresponding HPLC chromatograms of one positive result (e.g., CRF) and one negative result (e.g., LVB) are shown in Fig. 2. The maximal fluorescence signal was spread over several different HPLC-separated fractions, which could be an outcome of the HPLC separation not fully isolating the target chemical(s).

**Table 1:** Summary of HerboChip screening

Herbal extract	Abbreviation	Voucher specimen	Signal^a^
Crataegi Fructus^b^	CF	CF-1-2013	+
Cynanchi Paniculati Radix et Rhizoma	CPRR	CPRR-1-2013	++
Citri Reticulatae Semen	CRS	CRS-1-2013	+++
Dichroae Radix	DR	DR-1-2013	+++
Ecliptae Herba	EH	EH-1-2013	+++
Fagopyri Dibotrydis Rhizoma	FDR	FDR-1-2013	+++
Fritillariae Thunbergii Bulbus	FTB	FTC-1-2013	+++
Ganoderma^c^	GD	GD-1-2013	++
Ginkgo Folium	GKF	GKF-1-2013	+++
Gastrodiae Rhizoma	GR	GR-1-2013	+++
Gentianae Radix et Rhizoma^d^	GRR	GRR-1-2013	+++
Gleditsiae Sinensis Fructus	GSF	GSF-1-2013	+++
Kaki Calyx	KC	KC-1-2013	+++
Lycii Fructus	LF	LF-1-2013	++
Polygoni Multiflori Radix	PMR	PMR-1-2013	+++
Sanguisorbae Radix^e^	SR	SR-1-2013	+++
Sesami Semen Nigrum	SSN	SSN-1-2013	+

The plant species of herbs used match the Chinese Pharmacopeia (2010)

^a^The number of +s represents signal intensity. *n* = 5

^b^ *Crataegus pinnatifida* Bge var. *major* N. E. Br was used

^c^ *Ganoderma lucidum* (Leyss. Ex. Fr.) Karst was used

^d^ *Gentianarigescens* Franch was used

^e^ *Geranium scandens* (Hook f. et. Thoms) Hutch was used

The CM extracts that gave positive hits were further analyzed in cultured PC12 cells. The neuronal differentiation of PC12 cells was determined morphologically by measuring the length of neuritis. Three mammalian neurofilament subunits, NF68 (~68 kDa), NF160 (~160 kDa) and NF200 (~200 kDa), formed hetero-dimers that make the structural domain of neuritis [14, 15]. Application of NGF to cultured PC12 cells induced the expression of the neurofilaments in a dose-dependent manner (Additional file 1). This result agreed with previous reports [16], suggesting the successful establishment of the cell model.

**Fig. 3:**
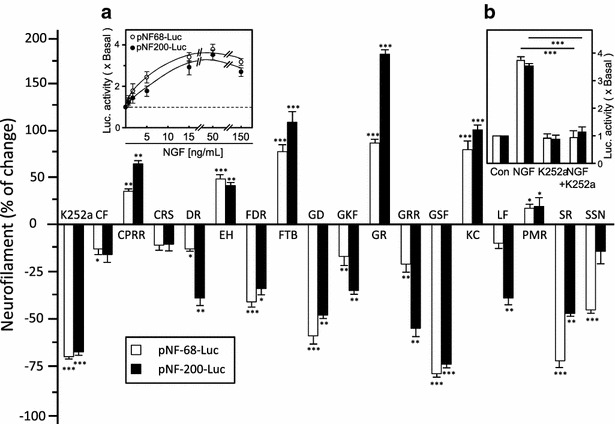
Herbal extracts in NGF-induced pNF-68/200 expression in PC12 cells. **a** PC12 cells were transfected with pNF-68/200-Luc and serum-starved for 3 h before co-treatment of 50 ng/mL of NGF and extract at 0.1 mg/mL. Cell lysates (50 μg) were subjected to a luciferase assay. **b** Activation by NGF is dose-dependent. K252a (100 nM, positive control) suppressed the NGF-induced response (*left inset*). Data are expressed as a percentage of change (mean ± SD, *n* = 4), where the values of NGF-added culture was set as 0. Statistical comparisons are between NGF alone (or its corresponding control) and the co-treatments, *P < 0.05, **P < 0.01, ***P < 0.001

Cell viability tests were performed to determine the maximum possible concentration of extracts that could be used before treatment. In general, the cells showed no morphological changes at concentrations under 0.1 mg/mL. The role of CM extracts that gave positive hits was revealed by interference with NGF in cultured PC12 cells and expressions of neurofilaments, NF68 and NF200, served as markers to evaluate the outcomes. The luciferase-reporters with NF68 or NF200 promoters (i.e., pNF-68-Luc and pNF-200-Luc) were transfected onto the cultures. Transfection efficiency was calculated at about 40–60 % using the β-galactosidase gene. CM extracts were applied with 50 ng/mL of NGF to transfected cells and incubated for 24 h. Cell lysates were collected for the luciferase assay. NGF induced promoter activity of NF68 and NF200 in transfected PC12 cells in a dose-dependent manner, which indicated the establishment of an appropriate cell model. The Trk receptor inhibitor K252a blocked NGF induced responses and served as the positive control (Fig. 3a). The effects of 17 herbal extracts on NGF-induced neurofilament expression are summarized in Fig. 3b. The extracts of Cynanchi Paniculati Radix et Rhizoma (CPRR), Ecliptae Herba (EH), Fritillariae Thunbergii Bulbus (FTB), Gastrodiae Rhizoma (GR), Kaki Calyx (KC) and Polygoni Multiflori Radix (PMR) significantly potentiated NGF responses. Many herbal extracts showed significant suppressive effects on NGF-induced neurofilament expression, including Dichroae Radix (DR), Fagopyri Dibotrydis Rhizoma (FDR), Ganoderma (GD), Ginkgo Folium (GKF), Gentianae Radix et Rhizoma (GRR), Gleditsiae Sinensis Fructus (GSF), Lycii Fructus (LF), Sanguisorbae Radix (SR) and Sesami Semen Nigrum (SSN).

**Fig. 4:**
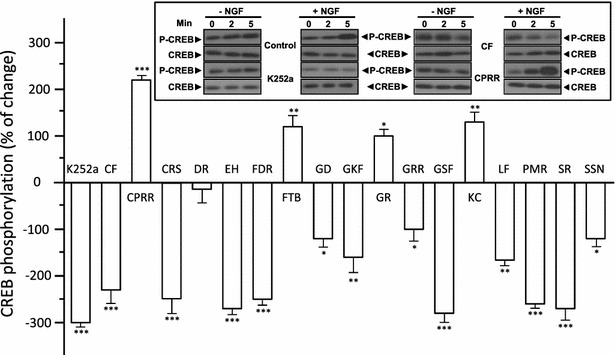
Herbal extracts in NGF-induced CREB phosphorylation in PC12 cells. PC12 cells were serum-starved for 3 h before co-treatment of 50 ng/mL of NGF and extract at 0.1 mg/mL, or K252a at 100 nM. Cell lysates (20 μg) were subjected to western blotting for phosphorylation analysis. NGF induced phosphorylation of CREB (P-CREB at ~42 kDa) in a time-dependent manner, which was altered by treatment of herbal extracts (*inset*). Band intensity was qualified. Data are expressed as a percentage of change (mean ± SD, *n* = 4), where the values of NGF added culture was set as 0. Statistical comparisons are between the NGF alone and the co-treatments: *P < 0.05, **P < 0.01, ***P < 0.001

CREB is involved in NGF-induced neuronal differentiation in cultured PC12 cells [14]. The phosphorylation of CREB was determined to further investigate the binding between the herbal fractions and NGF. PC12 cells were subjected to herbal extracts that gave positive-hits on the HerboChip arrays and the CREB phosphorylation level determined. The application of NGF induced phosphorylation of CREB (~43 kDa) in a time-dependent manner (Fig. 4). The effects of herbal extracts on CREB phosphorylation were tested as well as the effect on modulating NGF-induced phosphorylation. The extracts of CPRR, FTB, GR and KC potentiated CREB phosphorylation, which is in agreement with effects on neurofilament expression. Many of the HerboChip positive hits showed suppressive effects on NGF-induced CREB phosphorylation. The results of the neurofilament expression assay were similar to those obtained from the CREB phosphorylation investigations, confirming the NGF-binding effects of these CM extracts.

## Discussion

We screened over 300 herbal extracts and found 17 CM extracts that bound to NGF. These hits were tested in cultured PC12 cells for neurofilament expression and CREB phosphorylation. Both of these responses are downstream signals of Trk activation after NGF binding. The high percentage of positive hits is because the CM selected to manufacture the chips. The chosen 300 CM are all known herbs that are commonly used for their effects on brain functions. The herbal extracts had two regulatory influences on NGF-induced response in cultured PC12 cells, with binding of extracts to NGF either enhancing or reducing the binding of NGF to Trk A receptor. Four herbs, e.g., CPRR, FTB, GR and KC, showed parallel response in NGF-induced neurofilament expression and CREB phosphorylation. The discrepancy of the herbal extracts that showed contrasting responses of NGF-induced neurofilament and CREB phosphorylation, e.g., EH and PMR, could be accounted for by the potential that the extracts contain mixtures of chemicals affecting NGF signaling.

Increasing the bio-activity of NGF can facilitate neuron differentiation and neurite outgrowth [14]. CPRR, FTB and GR exhibited anti-nociceptive or sedation effects [16–19] and GR is a CM used for the treatment of mental disorder [20]. At least nine herbal extracts showed inhibition effects on NGF-induced responses. NGF-mediated CREB phosphorylation is regulated through the extracellular signal-regulated kinase (ERK) mitogen-activated protein kinases pathway [21]. The decreased phosphorylation after CM treatment was due to the extract blocking the ERK pathway, which is related to the Trk receptor. Anti-NGF agents have been proposed to cure osteoarthritis [22, 23] and CMs that inhibit NGF responses could be used to develop drugs for osteoarthritis.

Another potential application of this system would be the validation of active herbal fractions; however, this potential has been fully explored. The signals from the chip can be traced back to the isolated fraction(s) to identify the active chemicals binding to NGF. Fractions could be further separated using orthogonal HPLC separation and HerboChip screening to identify single chemicals that bind NGF. We are currently investigating and identifying the NGF binding chemicals from the extracts of CPRR, EH, FTB, GR, KC and PMR in our group. Although HerboChip cannot totally reflect the response between a target protein and herbal extracts, the platform is useful to provide information from a fast preliminary screening.

## Conclusion

The current result supported the applicability applicability of HerboChip for screening NGF binding components from herbal extracts.

## Additional file

10.1186/s13020-016-0107-8 Nerve growth factor (NGF) induces the differentiation of PC12 cells. (A): Image of PC12 cells treated with 50 ng/mL of NGF for 48 hours. Bar = 10 μm. (B): PC12 cells were serum-starved for 3 hours and then treated with 0.5 to 50ng/mL of NGF for 48 hours. The cell lysates (20 μg) were subjected to western blot for NF68 (~68 kDa), NF200 (~200 kDa) and GAPDH (~38 kDa). GAPDH served as a loading control, *n* = 4.

## Abbreviations

CM: Chinese medicine
NGF: nerve growth factor
CREB: cAMP response element-binding protein
PC12: pheochromocytoma
NF: neurofilament
CYP3A4: cytochrome P450 3A4
ERK: extracellular signal-regulated kinase
MAPKs: mitogen-activated protein kinases
MTT: 3-(4, 5-dimethyl-2-thiazolyl)-2, 5-diphenyl-2H-tetrazolium bromide
GAPDH: glyceraldehyde 3-phosphate dehydrogenase
