# Supplementary figures and images for: Indication of nerve growth factor binding components from herbal extracts by HerboChip: a platform for drug screening on a chip
Source: Chin Med. 2016 Jul 23;11:34. doi: 10.1186/s13020-016-0107-8 (PMC4958286; doi:10.1186/s13020-016-0107-8)

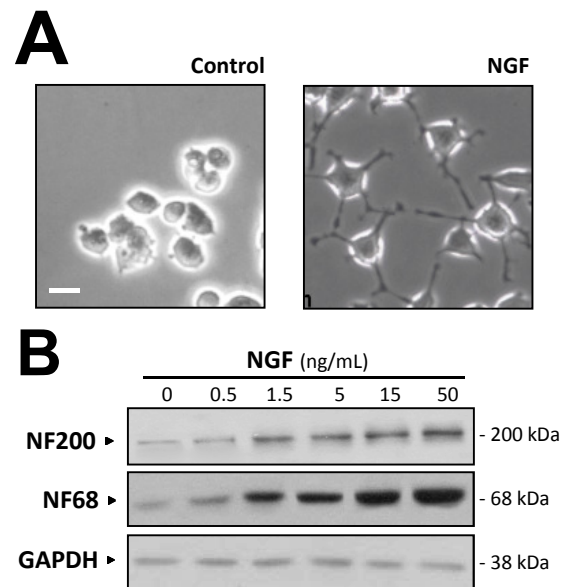

Supplement: Supplementary file 1 — 10.1186/s13020-016-0107-8 Nerve growth factor (NGF) induces the differentiation of PC12 cells. (A): Image of PC12 cells treated with 50 ng/mL of NGF for 48 hours. Bar = 10 μm. (B): PC12 cells were serum-starved for 3 hours and then treated with 0.5 to 50ng/mL of NGF for 48 hours. The cell lysates (20 μg) were subjected to western blot for NF68 (~68 kDa), NF200 (~200 kDa) and GAPDH (~38 kDa). GAPDH served as a loading control, n = 4. [file 13020_2016_107_MOESM1_ESM.pdf]
